# Using Location-Based Services Data to Map and Evaluate a Community Design Intervention to Increase Bicycling, Denver, Colorado

**DOI:** 10.5888/pcd21.230325

**Published:** 2024-10-17

**Authors:** Young Shin Park, Raymond J. King, Anu Pejavara, Kevin Hathaway, Jon Wergin, Cate Townley, Steph Leonard, John M. Williamson, Deborah A. Galuska, Janet E. Fulton

**Affiliations:** 1 Division of Nutrition, Physical Activity, and Obesity, Centers for Disease Control and Prevention, Atlanta, Georgia; 2 StreetLight Data, San Francisco, California; 3 Colorado Department of Public Health and Environment, Denver; 4 McKing Consulting Corporation, Atlanta, Georgia

**Figure:**
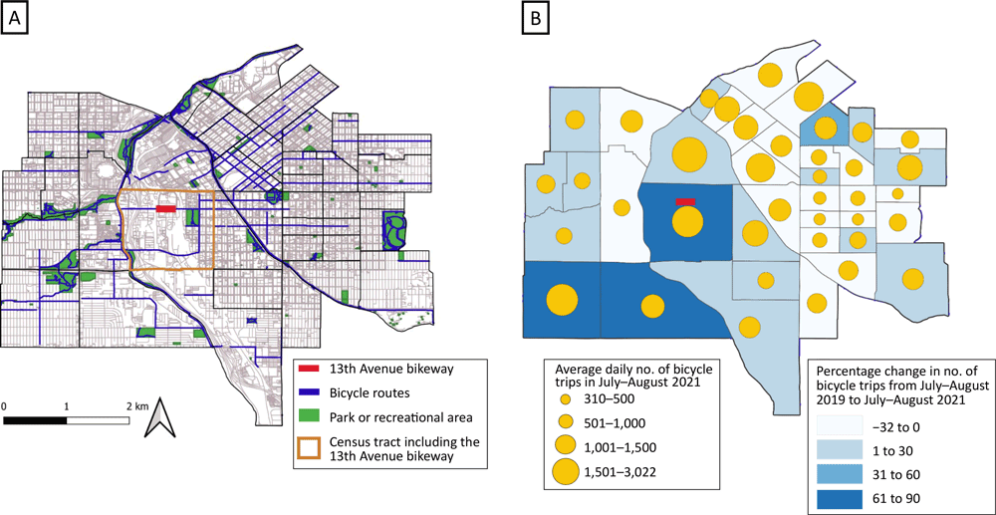
Maps can highlight connections of bicycle networks to amenities such as parks, which can facilitate physical activity through active transportation. Mapping bicycling volume after a community design intervention can provide information about the value of infrastructure investments to increase physical activity. A. Bicycle routes, parks, recreational areas, and the newly constructed 13th Avenue bikeway in downtown Denver, Colorado. B. Average daily bicycle volume in 2021 and percentage change in bicycle volume from July–August 2019 (before) to July–August 2021 (after) construction of the 13th Avenue bikeway in downtown Denver, Colorado.

## Purpose

Physical activity provides numerous health benefits (1). Community design interventions are an effective strategy for increasing physical activity through the enhancement of infrastructure such as paths, trails, and sidewalks, and the creation of activity-friendly routes to everyday destinations (2). The Centers for Disease Control and Prevention collaborates with partners, including state and local health departments across sectors (eg, transportation, parks), to increase physical activity by implementing community design interventions (3).

The evaluation of community design interventions is challenging. The intervention process often involves improving existing or constructing new infrastructure, which can take years to complete. Collecting evaluation data over long periods across communities can be labor-intensive and costly. On-the-ground counters are available to monitor walking and bicycling, but they require ongoing maintenance, such as battery replacement, and quality control checks to ensure counting accuracy (4). In addition, certain types of weather (eg, excessively hot or cold temperatures) are barriers to outdoor activity (5) and result in seasonal patterns of outdoor activity. To address this problem, evaluations must account for differences in physical activity according to season. Overcoming these challenges is important to efficiently evaluate community design interventions and promptly share findings with partners.

To evaluate a community design intervention intended to increase bicycling, we obtained data from StreetLight Data (6). Using location-based services data offers several advantages (7), such as not requiring on-the-ground data collection and enabling presentation of long-term trends for evaluation over multiyear periods. This study used location-based services data to meet 2 objectives: 1) evaluate changes in bicycling volume after construction of a bikeway in downtown Denver, Colorado, and 2) illustrate these changes through geovisualizations.

## Data and Methods

The 13th Avenue bikeway was constructed between July and October 2020 to enhance connectivity between the west and east sides of Denver. To assess whether the bikeway increased bicycle volume, we gathered data from several sources, including StreetLight Data (streetlightdata.com), the US Census Bureau, the Denver Regional Council of Governments (DRCOG), and OpenStreetMap (openstreetmap.org). We used StreetLight Data to assess the volume of bicycling before and after bikeway construction, from January 2019 through April 2022. StreetLight Data uses location-based data obtained from mobile devices (eg, cell phones) and applies proprietary machine-learning algorithms to aggregate data on transportation trips (8). A classification algorithm uses speed and distance, among other features, to identify bicycling trips (8). The StreetLight system identified bicycling trips on a defined segment of the 13th Avenue bikeway from mobile devices as they passed along that segment. Each trip represents bicycling-associated movement, not necessarily of a person, but of a bike. To define the study area, we used a 1-mile buffer around the 13th Avenue bikeway. The study area encompasses 39 census tracts; census tract boundary data are from 2020 Census TIGER data (9). We used DRCOG data (10) to visualize bicycle routes and OpenStreetMap (11) to visualize green areas such as parks.

We used QGIS 3.32 (QGIS Development Team) to create 2 maps. We created the first map to show locations of the 13th Avenue bikeway, bicycle routes, and green spaces, and outline the census tract in which the new bikeway was constructed. The second map was created to display the average daily bicycle volume and percentage change (before and after construction) in bicycling volume across census tracts. For the second map, we classified average daily bicycling volume in July and August 2021 into the following groups: 310 to 500, 501 to 1,000, 1,001 to 1,500, and 1,501 to 3,022 bicycling trips. We calculated percentage change as [(Bicycle trips in July–August 2021 minus Bicycle trips in July–August 2019) ÷ (Bicycle trips in July–August 2019)] × 100%. We classified percentage change into 4 groups: −32% to 0%, 1% to 30%, 31% to 60%, and 61% to 90%. We also graphed the average daily bicycle volume on the 13th Avenue bikeway during the study period.

### Difference of differences model

We conducted a difference-of-differences Poisson regression (PROC GENMOD, SAS version 9.4 [SAS Institute Inc]) to evaluate whether daily bicycle volume increased after bikeway construction (Appendix). The outcome was average daily bicycle volume in 2-month units. The main covariate represented bikeway preconstruction (value 0) versus postconstruction (value 1).

We modeled the difference of differences of average daily bicycle volume (postconstruction minus preconstruction) for the intervention tract minus the corresponding difference for the combined neighboring 39 census tracts. We included a variable for the intervention tract, the postconstruction versus preconstruction variable, and their interaction and controlled for seasonality (summer [May–October] vs winter [November–April]) and census tract. Testing for significance of the interaction variable allowed assessment of daily bicycle volume and accounted for a temporal trend. Significance testing for the Poisson regression model was conducted by using χ^2^ tests.

## Highlights

Average daily bicycling volume (Map B) increased significantly in the census tract that includes the 13th Avenue bikeway, from 937 trips before construction (January–February 2019 through May–June 2020) to 1,679 trips after construction (November–December 2020 through March–April 2022) (*P* < .001 for difference). The average number of daily bicycling trips on the bikeway was 325 trips before construction and 405 trips after construction (Figure).

**Figure:**
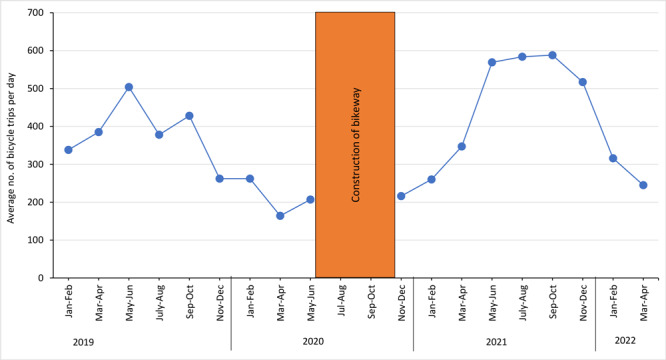
Average number of daily bicycle trips on the 13th Avenue bikeway, January–February 2019 to March–April 2022, Denver, Colorado. Construction took place from July through October 2020. Data source: StreetLight Data (https://www.streetlightdata.com).

**Table:** 

Measurement period	Average no. of bicycle trips
**2019**	
Jan-Feb	338
Mar-Apr	385
May-Jun	504
July-Aug	378
Sep-Oct	428
Nov-Dec	262
**2020**	
Jan-Feb	262
Mar-Apr	164
May-Jun	207
Jul-Aug	Construction
Sep-Oct	Construction
Nov-Dec	216
**2021**	
Jan-Feb	260
Mar-Apr	347
May-Jun	569
July-Aug	584
Sep-Oct	588
Nov-Dec	517
**2022**	
Jan-Feb	316
Mar-Apr	245

The Poisson regression model showed a 17% increase in daily bicycle volume (relative risk [RR] = 1.17; 95% CI, 1.13–1.21) in the combined other 39 tracts of metropolitan Denver (control area) versus a predicted increase of 88% (RR = 1.88; 95% CI, 1.46–2.42) in the intervention tract. Thus, compared with what we would expect were the bikeway not installed, we found 1.6 times more bicycling volume (RR = 1.60; 95% CI, 1.25–2.07) after installation of the new bikeway. A sensitivity analysis of the same months before (March 2019–April 2020) and after (March 2021–April 2022) construction showed essentially the same results as the original analysis (RR = 1.61 in sensitivity model vs RR = 1.60 in original model). The *P* value for a seasonality-by-construction variable was also not significant (.96).

## Action

We used location-based services data to visualize and evaluate changes in bicycling volume after construction of the 13th Avenue bikeway in Denver. We also compared bicycling patterns in the geographic areas surrounding the intervention. Our evaluation showed significant increases in bicycling trips after bikeway construction.

This study helps fill gaps identified by *The Community Guide* by prospectively evaluating community design interventions without collecting primary data (2). The use of a secondary data source, location-based services data, allowed us to assess the 3-year trend in bicycling volume on the bikeway and increased the efficiency of physical activity program evaluation. The location-based services data enabled continuous examination of changes in bicycling volume, eliminating the need for multiple cross-sectional data collections and saving time and human resources. Using continuously collected location-based services data allowed us to easily see whether bicycling volume varied by seasonal characteristics, such as air temperature (5).

Location-based services data have limitations. First, bicycling volume estimated from StreetLight Data approximates true bicycling activity. The comparison of StreetLight-estimated bicycling volume with on-the-ground counter estimates of bicycling volume has a margin of error of approximately 40 percentage points (12). Second, while we were unable to examine this factor, the margin of error in estimated bicycling volume may vary over time. Third, StreetLight-estimated bicycling may lack representativeness, although sociodemographic patterns of travelers (including bicyclists) compare favorably with patterns in surveys such as the National Household Travel Survey (13).

Mapping bicycling volume after a community design intervention can provide information for practitioners, researchers, and policymakers about the value of infrastructure investments to increase physical activity. The maps can also highlight connections of bicycle networks to amenities such as parks, which can facilitate active transportation.

Our findings illustrate how location-based services data, obtained from available mobile devices (eg, cell phones), can be used to efficiently evaluate local community design interventions to increase bicycling. The geovisualizations (and supporting analysis) show that bicycling volume increased after intervention implementation. The Colorado Department of Public Health and Environment will share the results of this pilot project with state and local partners, allowing them to easily communicate information on the effect of infrastructure investments for bicycling in Denver. These data will help Colorado plan for and evaluate future bicycle path investments as they expand the bicycle network. These types of visualizations may be useful to states and communities interested in evaluating community design interventions to increase walking or bicycling.

## Appendix Poisson Regression Analysis

### Model equations

The following equations were used to conduct the Poisson regression analysis. Let *t* denote the 2-month period of observation and log denotes natural logarithm. The response, *Y*, is distributed as a Poisson random variable. The base model using the one intervention zone containing the bikeway is:

Log(*Y_t_*) = β_0_ + β_1_
*X*_1_
*_t_* + β_2_
*X*_2t_

Where

*Y_t_* = daily biking volume,

*X*_1_
*_t_* = 1 for months May–October (summer); 0 for months November–April (winter),

*X*_2_
*_t_* = 1 for bikeway postconstruction; 0 for bikeway preconstruction.

The difference-in-differences model is:

log(*Y_t_*) = β_0_ + β_1_
*X*_1_
*_t_* + β_2_
*X*_2_
*_t_* + β_3_
*X*_3_
*_t_* + β_4_
*X*_4_
*_t_* + β_5_
*X*_5_
*_t_* + ¼ + β_43_
*X*_43_
*_t_*

where

*Y_t_* = daily biking volume,

*X*_1_
*_t_* = 1 for months May–October (summer); 0 for months November–April (winter),

*X*_2_
*_t_* = 1 for bikeway postconstruction; 0 for bikeway preconstruction.

*X*_3_
*_t_* = 1 for the one intervention zone containing the bikeway; 0 for other zones,

*X*_4_
*_t_* = *X*_2_
*_t_* ´ *X*_3_
*_t_* = 1 for intervention zone postconstruction; 0 otherwise

*X*_5_
*_t_*,⋯ , *X*_43_
*_t_* = binary indicators for each zone (fit with CLASS statement in SAS PROC GENMOD)

The difference-in-differences model including the seasonality by construction variable is:

log(*Y_t_*) = β_0_ + β_1_
*X*_1_
*_t_* + β_2_
*X*_2_
*_t_* + β_3_
*X*_3_
*_t_* + β_4_
*X*_4_
*_t_* + β_5_
*X*_5_
*_t_* + β_6_
*X*_6_
*_t_* + ¼ + β_44_
*X*_44_
*_t_*

Where

*Y_t_* = daily biking volume,

*X*_1_
*_t_* = 1 for months May–October (summer); 0 for months November–April (winter),

*X*_2_
*_t_* = 1 for bikeway postconstruction; 0 for bikeway preconstruction.

*X*_3_
*_t_* = 1 for the one intervention zone containing the bikeway; 0 for other zones,

*X*_4_
*_t_* = *X*_2_
*_t_* ´ *X*_3_
*_t_* = 1 for intervention zone postconstruction; 0 otherwise

*X*_5_
*_t_* = *X*_1_
*_t_* ´ *X*_2_
*_t_* = 1 for bikeway postconstruction and months May–October; 0 otherwise

*X*_6_
*_t_*, ¼ , *X*_44_
*_t_* = binary indicators for each zone (fit with CLASS statement in SAS PROC GENMOD).

### Model that included only the intervention census tract containing the bikeway

A Poisson regression model was fit (PROC GENMOD, SAS version 9.4) to evaluate whether daily bicycle volume increased after bikeway construction in the intervention census tract. This model used data only from the intervention census tract. This model showed an 80% increase in bicycle volume (relative risk = 1.80; 95% CI, 1.43–2.25) in the intervention tract.
